# Supplementary material for: The Synthesis and Evaluation of Aminocoumarin Peptidomimetics as Cytotoxic Agents on Model Bacterial E. coli Strains
Source: Materials (Basel). 2021 Sep 30;14(19):5725. doi: 10.3390/ma14195725 (PMC8510199; doi:10.3390/ma14195725)
Supplement: Supplementary file 1 [file materials-14-05725-s001.zip › materials-1338217-supplementary.pdf]

*Supplementary Materials*

# The Synthesis and Evaluation of Aminocoumarin Peptidomimetics as Cytotoxic Agents on Model Bacterial *E. coli* Strains

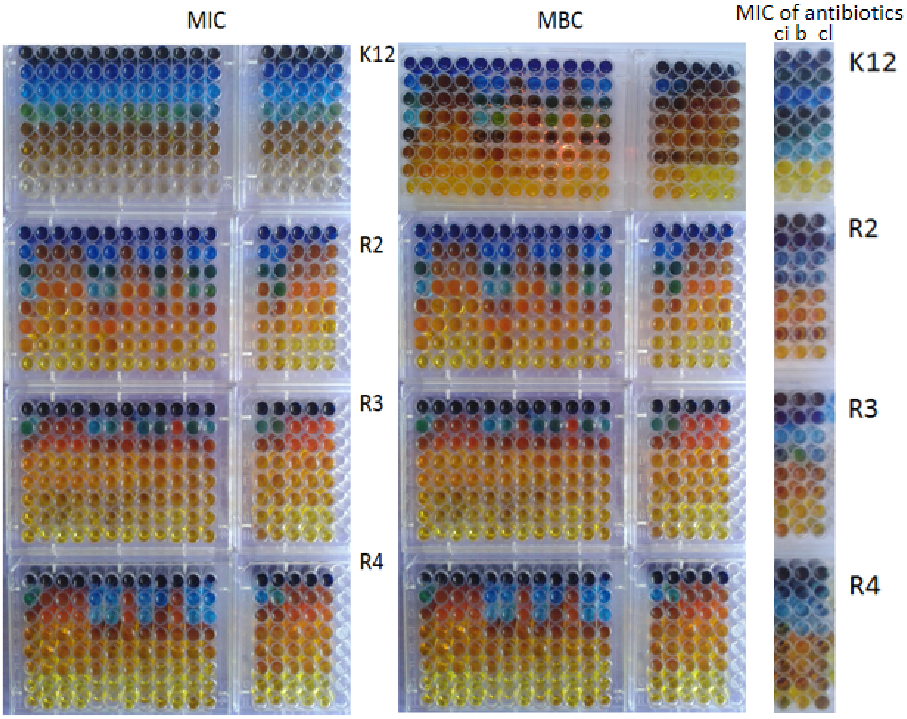


**Figure S1.** Examples of MIC and MBC on microplates with different concentration of studied compounds (μg/mL$^{-1}$). Resazurin was added as an indicator of microbial growth with K12, R2, R3, and R4 strains with tested 11 compounds, as described in Table 2. Additionally, examples of MIC with different strains K12, R2, R3, and R4 of studied antibiotics with ciprofloxacin (ci), bleomycin (b), and cloxacillin (cl) in (μg/mL$^{-1}$).

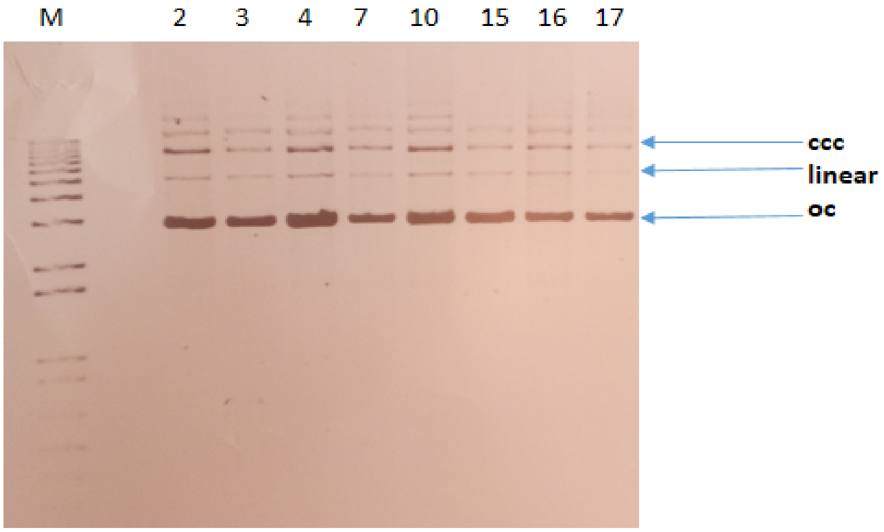

**Panela A**

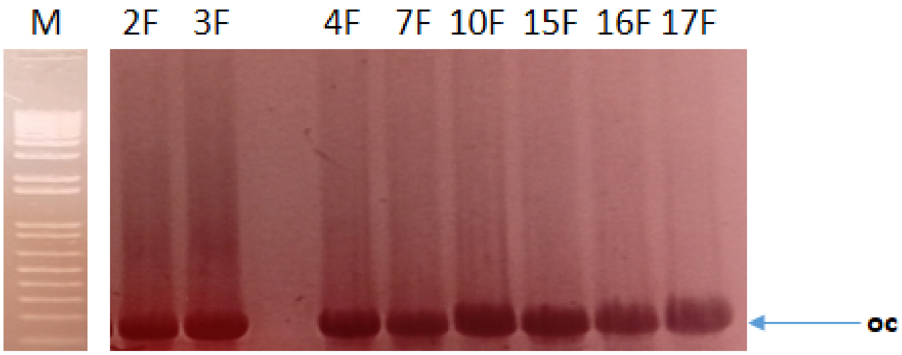


**Panela B**

**FigureS2.** An example of an agarose gel electrophoresis separation of isolated plasmids DNA on R4 strains modified with selected coumarin derivatives (Panel A) from 8 selected compounds, as shown in Figure 3, and digested with repair Fpg protein (Panel B). M = marker.

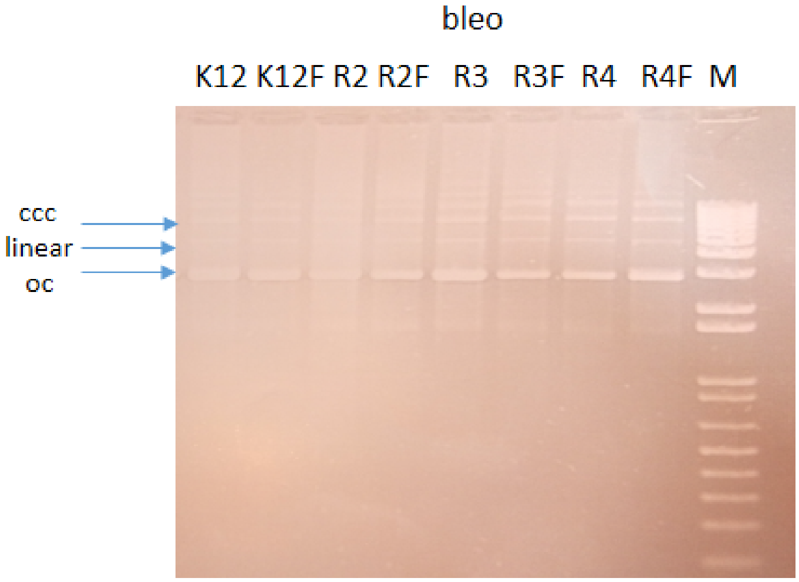


**Panela A**

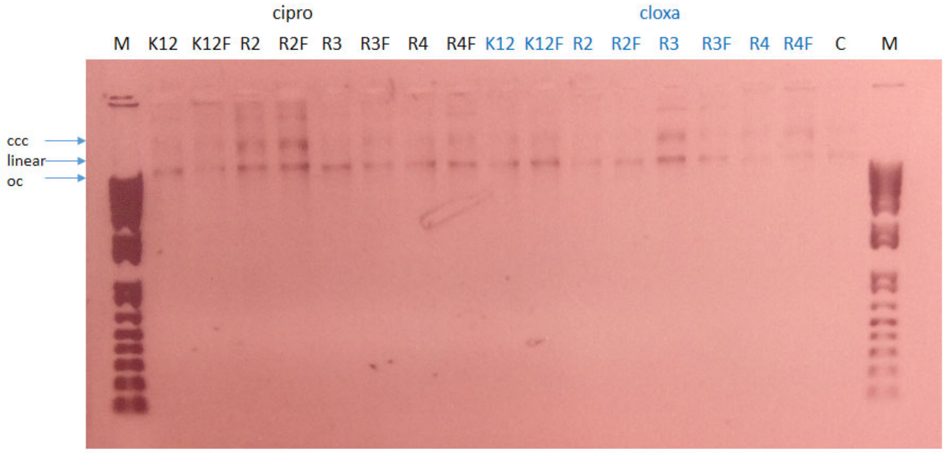


Panela B

**Figure S3.** Example of an agarose gel electrophoresis separation of isolated plasmids DNA from K12 and R4 strains modified with antibiotics: bleomycin (Panel A), ciprofloxacin, and cloxacillin (Panel B) digested (or not) with repair enzymes Fpg. M = marker.
